# 

**Published:** 1896-07

**Authors:** 


Marriages. At Trinity Church, Chicago, Ill., April 30th, by the Rev. John Rouse, Dr. A. H. Baker to Mrs. M. L. Treadwell.

At Philadelphia, on May 26, 1896, by Rev. F. W. Weiskotten, Dr. E. Mayhew Michener, of North Wales, Pa., to Miss Mamie C. Smith, of Philadelphia.

### SOCIETY PROCEEDINGS.

### PENNSYLVANIA STATE BOARD OF VETERINARY MEDICAL EXAMINERS.

THE second examination of the Board was conducted at Philadelphia on June 15th and 16th, at City Hall, commencing at 10 A.M. each day. The entire Board was present to conduct the various examinations as allotted to the several members.

Some twelve candidates presented themselves, representing the American, University of Pennsylvania, New York, and Toronto Veterinary Colleges.

The following applicants obtained the necessary percentage of 65 per cent. entitling them to the license of the Board: Drs. H. A. Christmann, Gilbert G. Drummond, John R. Mohler, H. D. Martien, of Philadelphia, graduates of the Veterinary Department of the University of Pennsylvania; Drs. A. W. Ormiston and Samuel Henry Johnston, of Philadelphia, graduates respectively of the American and the New York Veterinary Colleges; Drs. C. M. Heberton, Gwynedd, Pa.; Charles H. Miller, Duncannon, Pa.; James R. Mahaffy, of Wilmington, Del., graduates of the Veterinary Department of the University of Pennsylvania; and Dr. R. G. Rice, of Rome, Pa., graduate of the Ontario Veterinary College. One graduate of the Veterinary Department of the University of Pennsylvania and one from the Ontario Veterinary College failed to obtain the required average.

The Board elected officers for the ensuing year as follows: For President, Dr. W. Horace Hoskins, Philadelphia, Pa.; for Secretary-Treasurer, Dr. S. J. Harger, 205 N. Twentieth St., Philadelphia, Pa.

Prosecutions in a number of cases were ordered, and vigorous measures will be adopted in dealing with all violators of the several provisions of the Acts governing the practice of veterinary science in the Keystone State.

W. HORACE HOSKINS,
Secretary.

### PERSONAL.

Dr. J. H. Honan, professor of physiology at the McKillip Veterinary College, has resigned from the faculty.

Veterinarians Frank S. Allen and C. H. Zink, the latter a recent graduate of the Veterinary Department of McGill University, are considering propositions looking toward their connecting themselves with one of the Western veterinary colleges.

Veterinarians Osgood and Peters, of Boston, Mass., have gone into camp with the troops of the Bay State.

A number of changes will be made in the faculties of the Detroit and McKillip Veterinary Colleges the coming sessions of 1896–97.

Dr. H. D. Gill, of New York City, has been on the sick-list, and away from practice for a few days' rest and recuperation.

Samuel D. Bickel, V.M.D., of Norristown, was one of the graduates of the Medical Department of the University of Pennsylvania on June 11, 1896.

State Veterinarian Pearson, of Pennsylvania, was a recent visitor to Massachusetts on matters pertaining to the work of the State Veterinary Sanitary Board. Secretary Edge accompanied the State Veterinarian.

Dr. F. H. McCarthy, of Pottsville, has recently suffered from appendicitis, and was operated upon successfully at the hospital at his home.

Dr. N. H. Fegley, of Pottsville, successfully passed the civil-service examination of the Bureau of Animal Industry, and has been assigned for duty at Jersey City.

Dr. H. D. Gill, of New York City, has successfully passed the civil-service examination and been appointed to a position as Inspector in the Bureau of Animal Industry, with directions to report at Chicago for duty.

Dr. Cecil French, of Washington, D. C., has just completed the purchase of ground in one of the suburbs, where he will at once erect a canine infirmary, consisting of a number of wards with runs attached. An isolated contagious ward will be one of the leading features; also, an operating-room, shampooing-room, feed-room, etc. The whole building will be heated by a hot-water plant. A boarding department and canine ambulance will complete the undertaking.

Dr. M. E. Knowles has severed his connection with the Marcus Daly ranch at Hamilton, Montana.

Dr. W. L. Rhoads, of Lansdowne, Pa., has placed plans in the hands of contractors for the construction of a commodious veterinary hospital. It will be two stories in height, and in addition to office, pharmacy, elevator, harness-room, etc., there will be eight single and two box-stalls on the first floor.

Prof. A. Liautard, of New York, continues to remain abroad, and will probably not return to America until the fall.

Dr. John S. Buckley, honor-man at the recent graduating exercises of the American Veterinary College, has been appointed an instructor in veterinary science at the Maryland Agricultural College.

Dr. L. P. Cook, of Cincinnati, late Professor of Surgery in the Ohio Veterinary College at Cincinnati, has successfully passed the civil-service examination of the Bureau of Animal Industry, and been appointed an inspector and assigned for duty at the port of New York.

Dr. Huidekoper is on a visit to his brother at Meadville, Pa. A physical examination lately made revealed the presence of fluid in his chest, necessitating his retiring for a time from his practice, and compelling him to seek a different climate.

Dr. Edward N. Leavy, valedictorian of the Class of '96, American Veterinary College, has opened a well-equipped and located dog-hospital at Lexington Avenue and Sixty-first Street, New York City. The doctor is making a specialty of canine diseases.

Dr. Martin has bought the practice of Dr. James Hamill, deceased.

### AMERICA OR CANADA.

WANTED to know of a genuine opening for an experienced M.R.C.V.S. at either of the above places. Anyone giving reliable information of a suitable town will be rewarded. Informants should state the name and population of the town, and whether there is or has been a veterinary surgeon there, also the nature and extent of the practice likely to be done. Address, 2505, V.R., 20 Fulham Road, London, S.W., England.

